# Supplementary material for: Ultra-durable superhydrophobic cellular coatings
Source: Nat Commun. 2023 Sep 23;14:5953. doi: 10.1038/s41467-023-41675-y (PMC10517967; doi:10.1038/s41467-023-41675-y)
Supplement: Supplementary file 1 — Supplementary Information [file 41467_2023_41675_MOESM1_ESM.pdf]

Supplementary Information

Ultra-durable superhydrophobic cellular coatings

Wancheng Gu, Wanbo Li*, Yu Zhang, Yage Xia, Qiaoling Wang, Wei Wang, Ping Liu, Xinquan Yu, Hui He, Caihua Liang, Youxue Ban, Changwen Mi, Sha Yang, Wei Liu, Miaomiao Cui, Xu Deng*, Zuankai Wang*, Youfa Zhang*

*Corresponding author. E-mail: wanboli@sjtu.edu.cn, dengxu@uestc.edu.cn, zk.wang@polyu.edu.hk, yfzhang@seu.edu.cn

Table of Contents

Supplementary Discussion

1. Finite element (FE) modeling of physical shield

2. Simulation of chemical bridge

3. Theoretical models for optimizing the coating strength

Supplementary Figures 1-30

Supplementary Tables 1-3

Supplementary Discussion

1. Finite element (FE) modeling of physical shield

We conducted FE modeling to reveal the stress distribution on the coating surface under normal load. The geometric model was simplified as a single cell loaded in the matrix and was designed in Pro/E Wildfire 5.0 software. The FE model was further established with refined mesh by using multipurpose FE software ANSYS Workbench. The elasticity modulus of the cell and matrix was set to 80 and 5 GPa, respectively. The vertical and horizontal loads were set to 1 and 0.3 mN, respectively, corresponding to a friction coefficient of 0.3. All the materials were considered ductile, that is, tolerating more than 5% elongation before breaking at stretching, so that the material does not yield or fail under actual service loads. For the simulation, the von-Mises theory was utilized to make sure that the materials are safe as long as the maximum distortion energy per unit volume kept smaller than its critical distortion energy per unit volume. The stress state at a particular location can be characterized by three principal stresses σ_1, σ_2, and σ_3, which are in the order of magnitude and sign $\sigma_1 > \sigma_2 > \sigma_2$, left-hand side of the equation referred to the von-Mises stress σ_e, then for yield, adjust it by an appropriate factor of safety *f.s.*

$$\sigma_e = \sqrt{\frac{[(\sigma_1-\sigma_2)^2+(\sigma_2-\sigma_3)^2+(\sigma_1-\sigma_3)^2]}{2}} \geq \frac{S_y}{f.s.} \quad (1)$$

Namely, the maximum Von-Mises stress induced at a critical tensile point under triaxial combined stress ≤ permissible yield stress S_y considering safety factor f.s.

According to Eq. (1), the equivalent von-Mises stress is used to determine whether the ductile microstructures will yield or not when subjected to external loads. The simulation results demonstrate that the cell bore major equivalent von-Mises stress, provided a strong physical shield that reinforces the coating mechanical stability (Supplementary Fig. 1a).

2. Simulation of chemical bridge

An appropriate density of covalent bonds is crucial for balancing the mechanical stability and superhydrophobicity of the coating. To understand such influence, we performed periodic Density Functional Theory (DFT) simulations (Vienna Ab initio Simulation Package, Austria) to calculate the matrix binding energy and water adsorption energy with the diatomite surface according to the method in the previous report[6]. As illustrated in Supplementary Fig. 1b and 1c, the calculation model was composed of a silica surface with part of the hydroxy groups being silanized. The

generalized gradient approximation functional was adopted with the Perdew−Burke−Ernzerh of exchange−correlation description[7]. The DFT + vdW method was used to account for the van der Waals interactions[8]. The structure was optimized until the force on each atom was smaller than 0.05 eV/Å. The two-dimensional Brillouin zone was sampled with a 5 × 5 × 1 mesh according to the Monkhorst−Pack method[9]. The SiO_2 surface was a 2 × 2 supercell of α-quartz SiO_2 (0001) with hydroxyl group terminals. To avoid the interlayer interaction, we added a vacuum of more than 10 Å between the slabs.

Supplementary Figure 1d shows the change of matrix binding energy energies and water adsorption energies as a function of surface covalent bond density. Increasing the covalent bond density significantly enhances both the binding energy with matrix and adsorption energy with water, suggesting a tradeoff between bonding strength and hydrophobicity. An optimum regime of the covalent bond density emerges at ~50%, promising both sufficient silane groups for superhydrophobic functionality and adequate reactive hydroxy groups for chemical bridging. The results are consistent with the experimental data.

3. Theoretical models for optimizing the coating strength

The mechanical strength of a bulk-phase coating can be predicted based on the Griffith-Irwin-Orowan theory, for guiding the design of durable superhydrophobic coating. In this model, the cells act as a reinforcement phase to improve the elasticity modulus of the matrix, which can be expressed as the following equation:

$$\sigma_c = \left(\frac{2E\gamma_p}{\pi a}\right)^{1/2} \tag{2}$$

where E is the elasticity modulus of coating, γ_p is the total plastic work before the coating breaking, and a is the crack length. The elasticity modulus of composite coatings can be represented by a generalized rule of the form as below:

$$E = E_1V_1 + E_2V_2 \tag{3}$$

where E, E_1, and E_2 are the elasticity modulus of coating, cells, and matrix, respectively, and V_1 and V_2 are the volume fraction of cells and matrix, respectively. Therefore, Eq. (2) can be deduced as:

$$\sigma_c^2 - \frac{2\gamma_p(E_1-E_2)}{\pi a\rho_1}\alpha - \frac{2\gamma_p E_2}{\pi a} = 0 \tag{4}$$

Where, α is the mass fraction of cell (i.e., cell content), and ρ_1 is the density of cells.

Further consider the influence of the cell content, that is, the increment in cell content

introduces more cracks and reduces the plasticity. The relationship between plastic work γ_p , (integration of the plastic deformation region under the stress-strain curve) and cell content was obtained by the curve fitting (Supplementary Fig. 9a):

$$\gamma_p = k_1\alpha^2 + m_1\alpha + n_1 \tag{5}$$

By substituting Eq. (5) into Eq. (4), the relationship between the coating fracture strength and cell content can be expressed as:

$$\sigma_c^2 = \frac{2(E_1 - E_2)(k_1\alpha^3 + m_1\alpha^2 + \alpha)}{\pi a \rho_1} + \frac{2E_2(k_1\alpha^2 + m_1\alpha + n_1)}{\pi a} \tag{6}$$

that is:

$$\sigma_c^2 \propto -\alpha^3 + \mathrm{A}\alpha^2 + \mathrm{B}\alpha \tag{7}$$

where A and B are the correlation coefficients that are inversely proportional to the crack length and increase linearly with the elasticity modulus difference between the cell and matrix.

Similarly, the relationship between the coating stress and the covalent bond density agrees with the Griffith-Irwin-Orowan theory. In this model, the interface between a silanized cell and matrix was assumed to be the initial cracks due to the relatively weak interfacial strength. The growth of cracks under external load drives the bulk coating failure. Generally, the decrease in the covalent bond density β in such an interface translates the principal cohesion force from chemical bonding to physical absorption, causing the increment in crack length and deterioration in the plastic work γ_p. Assuming that chemical groups distribute uniformly on the cell surface, the relationship between crack length and covalent bond density can be expressed as:

$$a = l(1 - \beta) \tag{8}$$

And the relationship between plastic work and covalent bond density was obtained by the curve fitting (Supplementary Fig. 9b):

$$\gamma_p = k_2(\beta + m_2)^2 + n_2 \tag{9}$$

By substituting Eq. (8) and (9) into Eq. (2), the relationship between the coating strength and covalent bond density can be expressed as:

$$\sigma_c^2 = -\frac{2Ek_2(\beta + m_2)^2 + 2n_2E}{\pi l(\beta - 1)} \tag{10}$$

that is:

$$\sigma_c^2 \propto \frac{(\beta + \mathrm{C})^2}{\beta} \tag{11}$$

where parameter C is a factor for calibrating the heterogeneity of covalent bonds and cell distribution.

We further analyzed the influence of the cell size on coating strength as follows. The mechanical strength of the cellular coating with different cell diameters *d* (here, *d* is the average diameter characterized by laser particle size analyzer, Mastersizer 3000, UK) can also be predicted based on the Griffith-Irwin-Orowan theory. First, V_1 and V_2 (the volume fraction of cells and matrix) can be respectively expressed as follow:

$$V_1 = \frac{\pi(d_1^2 h_1 + d_2^2 h_2 + d_3^2 h_3 + \cdots + d_N^2 h_N)}{4V} = \frac{\pi(i_1 d_1^3 + i_2 d_2^3 + i_3 d_3^3 + \cdots + i_N d_N^3)}{4V} \tag{12}$$

$$V_2 = 1 - \frac{\pi(i_1 d_1^3 + i_2 d_2^3 + i_3 d_3^3 + \cdots + i_N d_N^3)}{4V} \tag{13}$$

where d_1, d_2, d_3 …d_N are the diameter of each cell, h_1, h_2, h_3 …h_N are the height of each cell, and i_1, i_2, i_3 …i_N are the correlation coefficient between diameter and height, respectively. Therefore, Eq. (3) can be deduced as:

$$\begin{aligned} E &= E_1 \frac{\pi(i_1 d_1^3 + i_2 d_2^3 + i_3 d_3^3 + \cdots + i_N d_N^3)}{4V} + E_2\left(1 - \frac{\pi(i_1 d_1^3 + i_2 d_2^3 + i_3 d_3^3 + \cdots + i_N d_N^3)}{4V}\right) \\ &= E_2 + \frac{\pi}{4V}(E_1 - E_2)(i_1 d_1^3 + i_2 d_2^3 + i_3 d_3^3 + \cdots + i_N d_N^3) \\ &= E_2 + \frac{\pi}{4V}(E_1 - E_2)(i_1 j_1^3 + i_2 j_2^3 + i_3 j_3^3 + \cdots + i_N j_N^3) d^3 \\ &= D + M d^3 \end{aligned} \tag{14}$$

where j_1, j_2, j_3 …i_N are the correlation coefficient between the diameter of each cell and the average diameter. As the initial crack is the interface between the cell and matrix, the crack length *a* is in direct proportion to the cell diameter *d*:

$$a = k_3 d \tag{15}$$

By substituting Eq. (14) and (15) into Eq. (2), the relationship between the coating fracture strength and cell diameter *d* can be expressed as:

$$\sigma_c^2 = \frac{2\gamma_p}{\pi k_3 d}(D + M d^3) \tag{16}$$

that is:

$$\sigma_c^2 \propto P d^2 + \frac{Z}{d} \tag{17}$$

where parameters P and Z are the contribution factors illustrating the reinforcement effect of the cell and the deterioration effect induced by the initial crack in the interface of the cell and matrix, respectively. In other words, when the cell diameter is relatively small, the reinforcement effect of

the cell plays a dominant role. On the contrary, when the cell diameter is relatively large, the deterioration effect of the crack determinates the coating strength.

The respective fit of Eq. (7), (11) and (17) with the experimental data points in Fig. 1e,f and Supplementary Fig. 10, demonstrated a high consistency between the theoretical calculation and experimental results, suggesting the feasibility of the Griffith-Irwin-Orowan theory for predicting the mechanical strength of superhydrophobic coatings.

Supplementary Figures

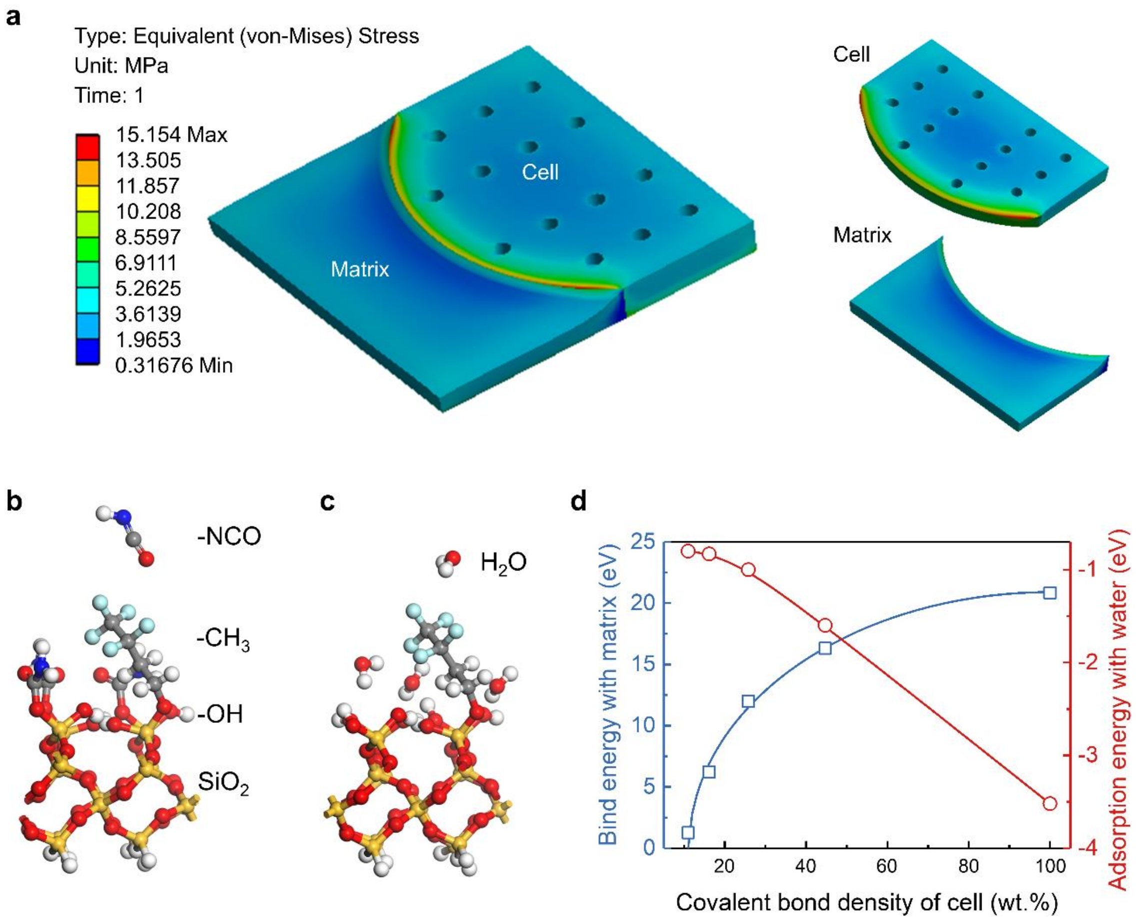

Supplementary Fig. 1 Modeling the mechanical and chemical properties of the cell. a, FE simulation of the stress distribution in the cellular coating. The cell bears the main equivalent von-Mises stress and protects the matrix and seeds from damage. **b**, **c**, DFT molecular model for calculating the binding energy between matrix molecules with cell surface via either physical adsorption or chemically bonding (**b**) and the binding energy of water molecules with cell surface via physical adsorption (**c**). **d**, Change in the matrix binding energy and water absorption energy with cell surface as a function of covalent bond density.

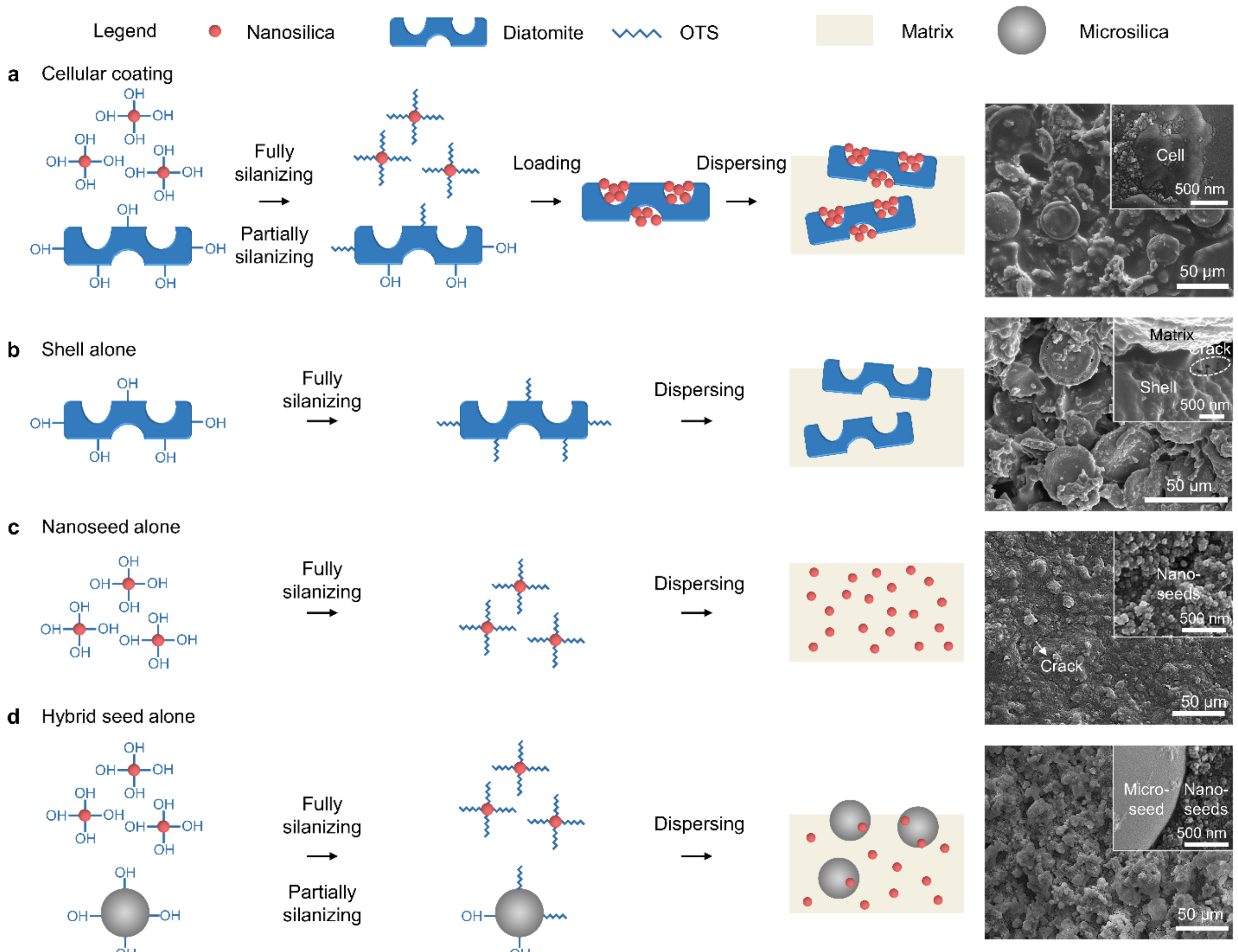

Supplementary Fig. 2 Schematic illustration of the preparation and the corresponding SEM images of the resultant coatings: a, cellular coating, **b**, shell-alone coating, **c**, nanoseed-alone coating, and **d**, hybrid seed-alone coating.

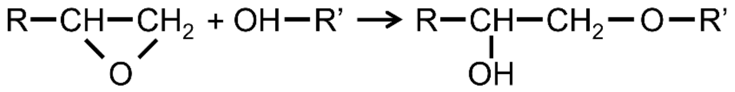

(1)

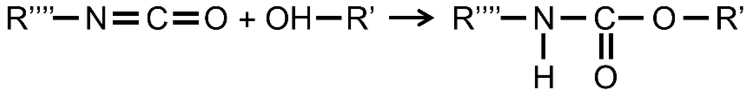

(2)

Supplementary Fig. 3 Chemical reaction for forming chemical bonds between matrix and cells. The covalent bonds formed through the condensation reactions between the -OH group on the cell surface and the reactive group (e.g., epoxy groups and isocyanate groups).

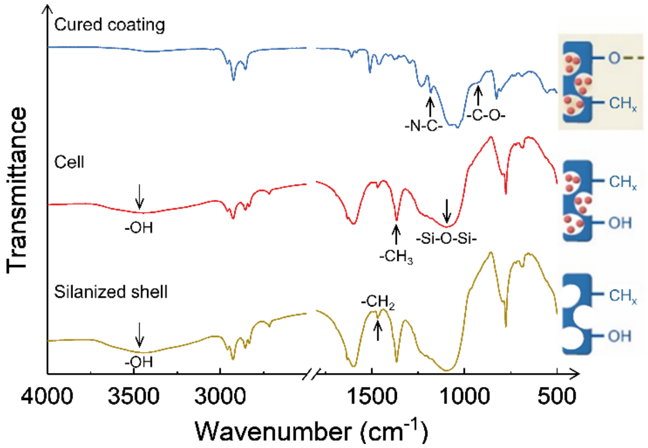

Supplementary Fig. 4 Chemical bond characterization. FTIR spectra of the silanized shells, impregnated cells after silanization, and cured cellular coating. The partially silanized cell surface presented absorption peaks for -OH and $-CH_x$ groups, which kept stable after the cells were dispersed in the epoxy matrix. After curing, the remaining -OH groups on the cell thoroughly reacted with active groups in the matrix, forming strong covalent bonds.

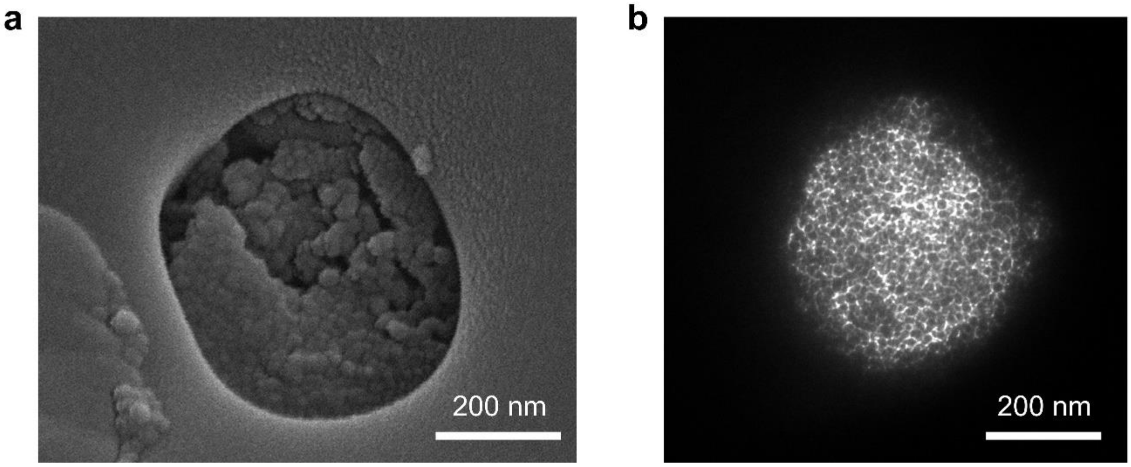

Supplementary Fig. 5 Nanoseed loading. SEM (**a**) and TEM (**b**) images of the nanoseeds in the shell.

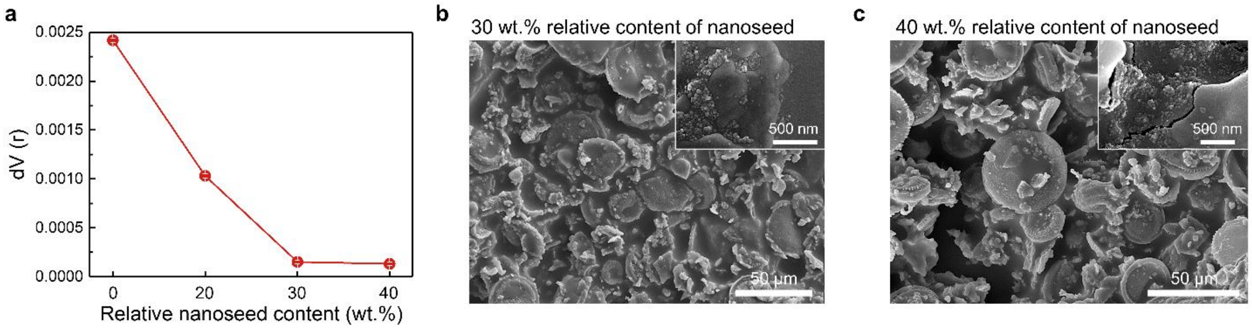

Supplementary Fig. 6 Cell preparation and optimization. a, The remaining pore volume of the shell as a function of the seed/shell mixture ratio, as was analyzed by Brunauer−Emmett−Teller analyses (ASAP 2460, Micromeritics, USA). The remaining pore volume reaches a plateau at 30 wt.%, which indicates the saturate loading fraction. The errors represent the standard deviations from at least three independent experiments. **b**, **c**, Surface morphologies of cellular coatings with relative seed content of 30 wt.% (**b**) and 40 wt.% (**c**). At saturate point, all the seeds were stored in the diatomite shell which results in a continuous interface with the matrix. In contrast, excessive loading of fully silanized seed destroys phase compatibility, which leads to crack formation.

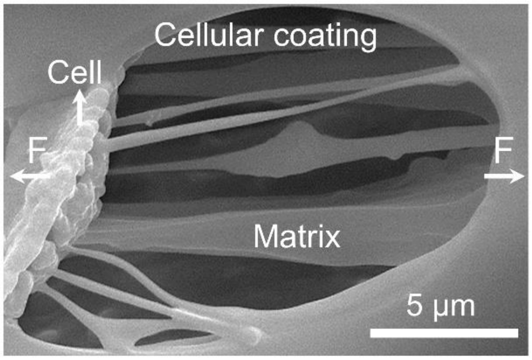

Supplementary Fig. 7 Solid adhesion between the cell and matrix. Morphology of the cellular coating after stretching at the yield point.

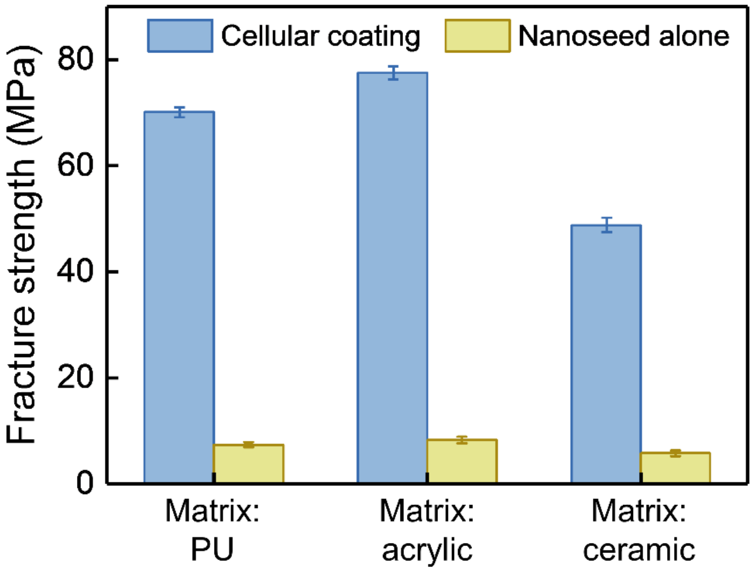

Supplementary Fig. 8 Fracture strength measurements. Comparison of the fracture strength of cellular coatings and nanoseeds coatings in different matrixes, including PU resin, polyacrylic acid resin, and ceramic matrix. The errors represent the standard deviations from at least three independent experiments.

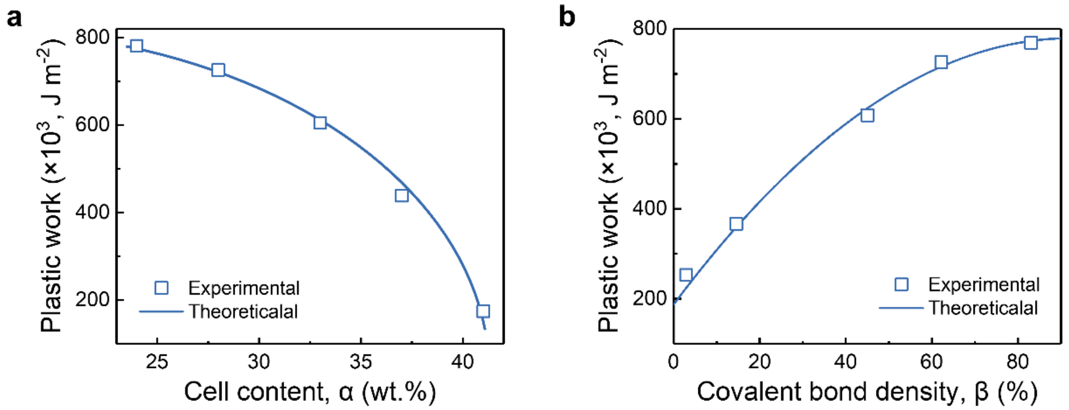

Supplementary Fig. 9 Plastic work measurements. Relationship between plastic work and cell content α (**a**) and covalent bond density β (**b**) of cellular coatings.

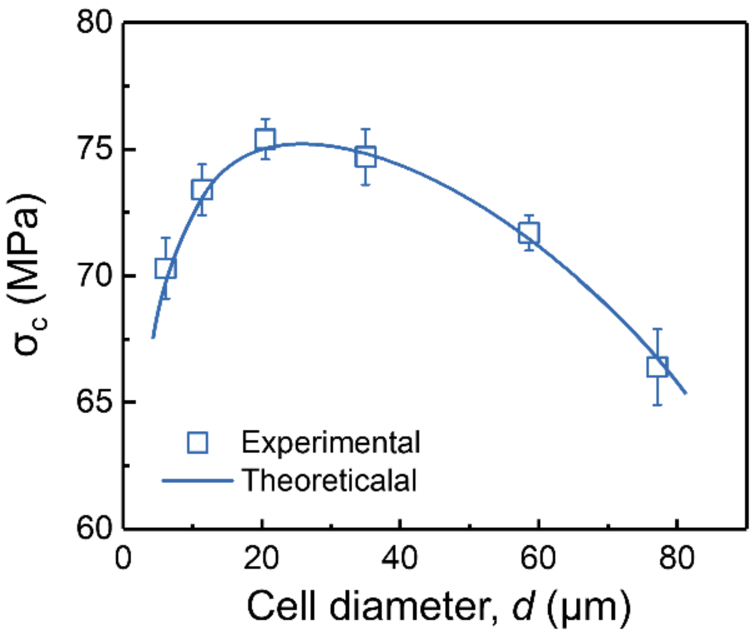

Supplementary Fig. 10 Influence of cell size on the coating fracture strength. Change of the coating fracture strength σ_c as a function of the cell diameter d. The errors represent the standard deviations from at least three independent experiments.

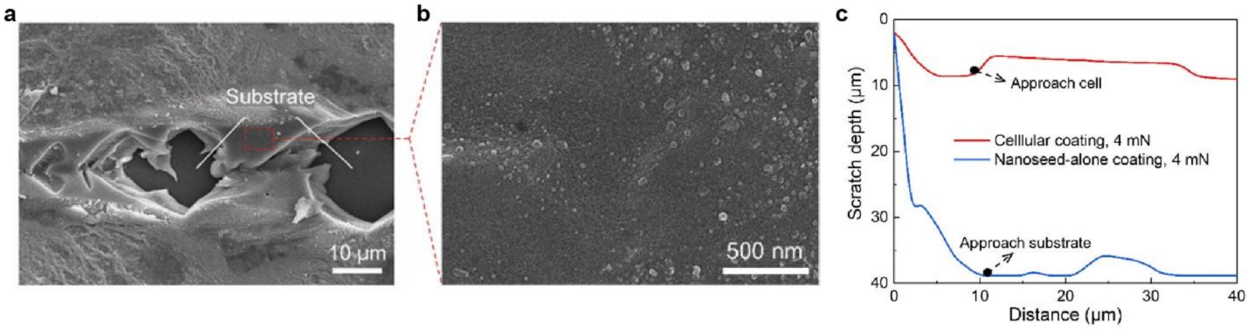

Supplementary Fig. 11 Smooth surface of nanoseed-alone coating after micro-scratching. a,**b**, SEM images of the nanoseed-alone coating (a) and zoom-in view of few nanoseeds (b) after micro-scratching under a load of 4 mN. **c**, Change of the scratch depth with scratching distance for the cellular coating and nanoseed-alone coating under 4-mN load.

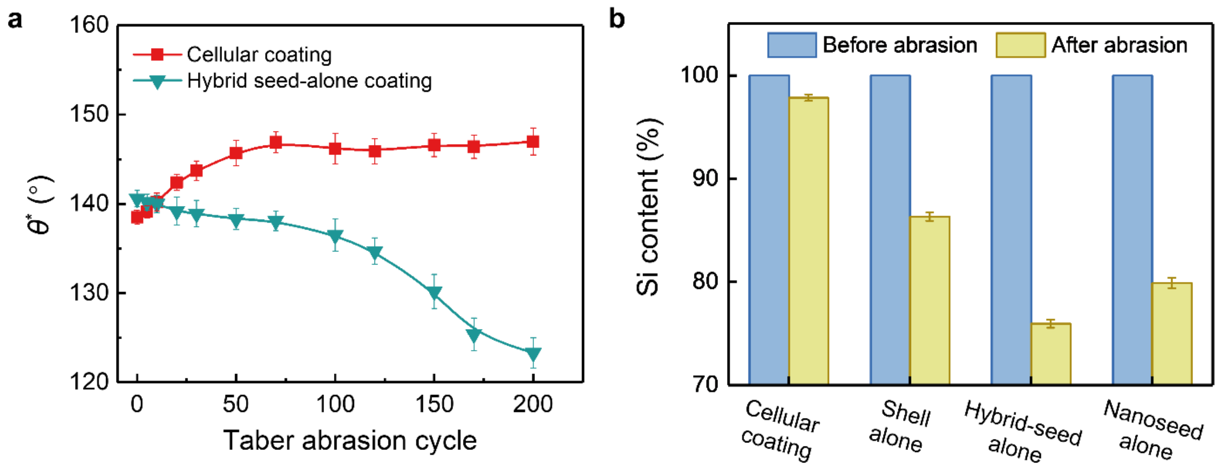

Supplementary Fig. 12 Validation of nanoseed release by measurements of water repellency and Si content after Taber abrasion test based on a cellular coating with a special design (cellular coating with hydrophilic shells and hydrophobic seeds). In such a cellular coating, the water repellency was only determined by the hydrophobic nanoseeds. The hybrid seed-alone coating was used for comparison. **a**, Water repellency of the coatings along with the repeated abrasion, the cellular coating demonstrated an increase in the water repellency and kept stable thereafter, whereas hybrid-seed alone coating lost the water repellency rapidly. **b**, Comparison of Si content reduction of different coatings after Taber abrasion. Both measurements demonstrated the release of nanoseeds well. The errors represent the standard deviations from at least three independent experiments.

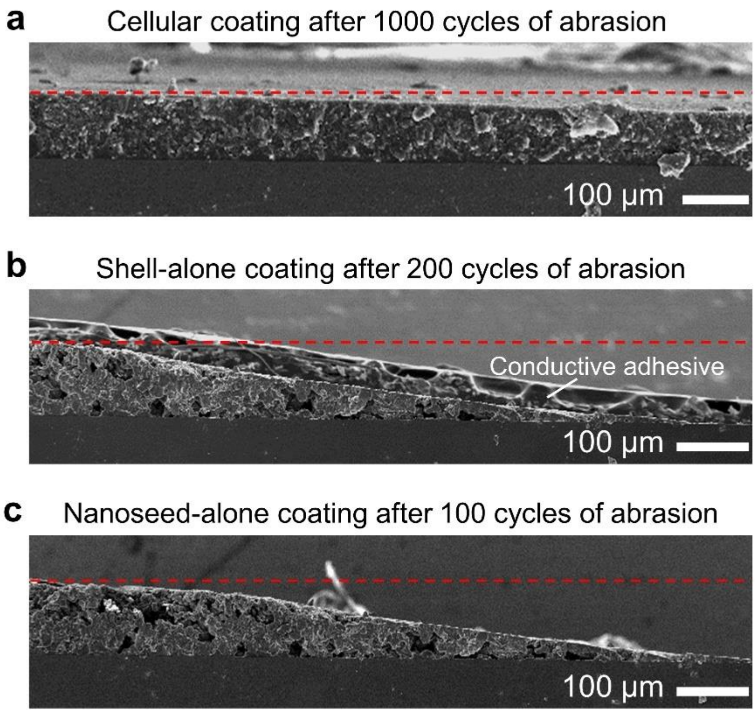

Supplementary Fig. 13 SEM images showing the thickness reduction of different coatings after Taber abrasion (load: 1 kg). a, Cellular coating, **b**, shell-alone coating, and **c**, nanoseed-alone coating after Taber abrasion.

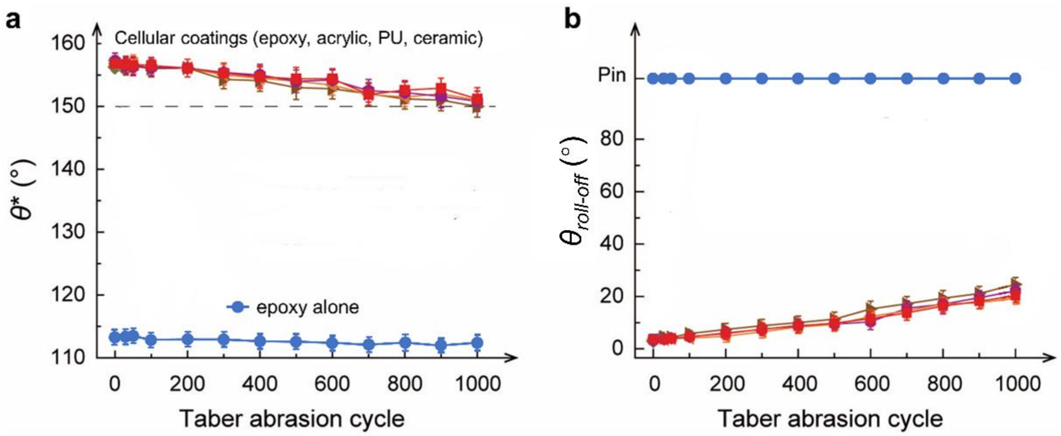

Supplementary Fig. 14 Comparison of cellular coatings and matrix-alone coatings. a, b, Evolution of water contact angles (a) and roll-off angles (b) of different coatings during Taber abrasion under 1-kg load. The errors represent the standard deviations from at least five independent experiments.

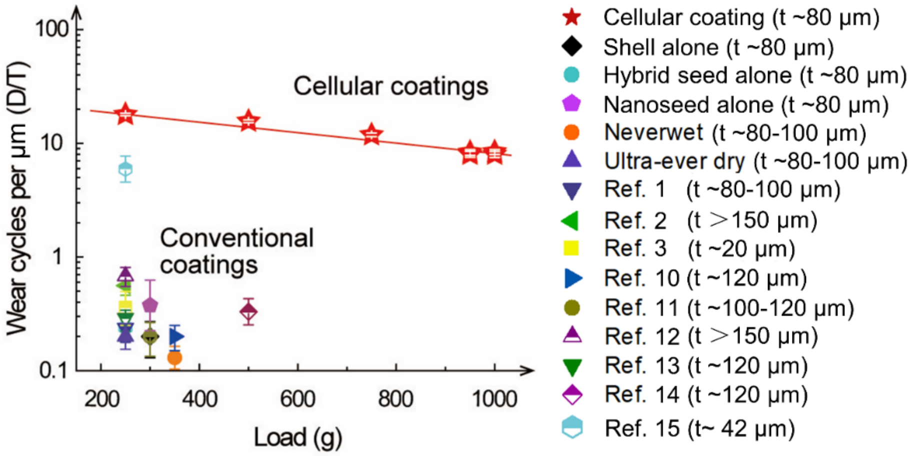

Supplementary Fig. 15 Comparison of the mechanical stability of the cellular coating with that of control coatings and the state-of-art coatings with various thicknesses *t*. The errors represent the standard deviations from at least three independent experiments.

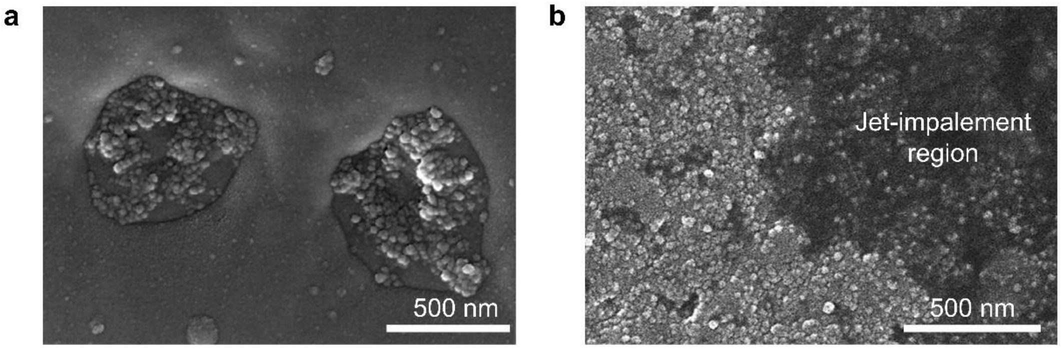

Supplementary Fig. 16 SEM images showing the nanoseeds on the cellular coating (a) and nanoseed-alone coating (b) after water jet impalement for 4 s (*We* ~44444). Note that the nanoseeds in the cell pores were protected from impalement, whereas the nanoseeds on the surface of the nanoseed-alone coating were totally removed from the impalement region.

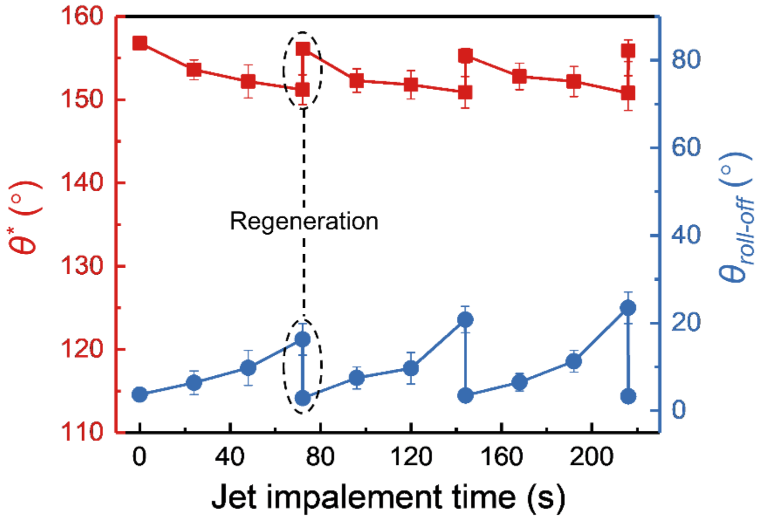

Supplementary Fig. 17 Evolution and regeneration of water repellence of the cellular coating during the water jet impalement (*We* ~44444). The jet impalement gradually degraded the superhydrophobicity, which can be regenerated via a gentle abrasion to release the nanoseeds in the cells. The errors represent the standard deviations from at least five independent experiments.

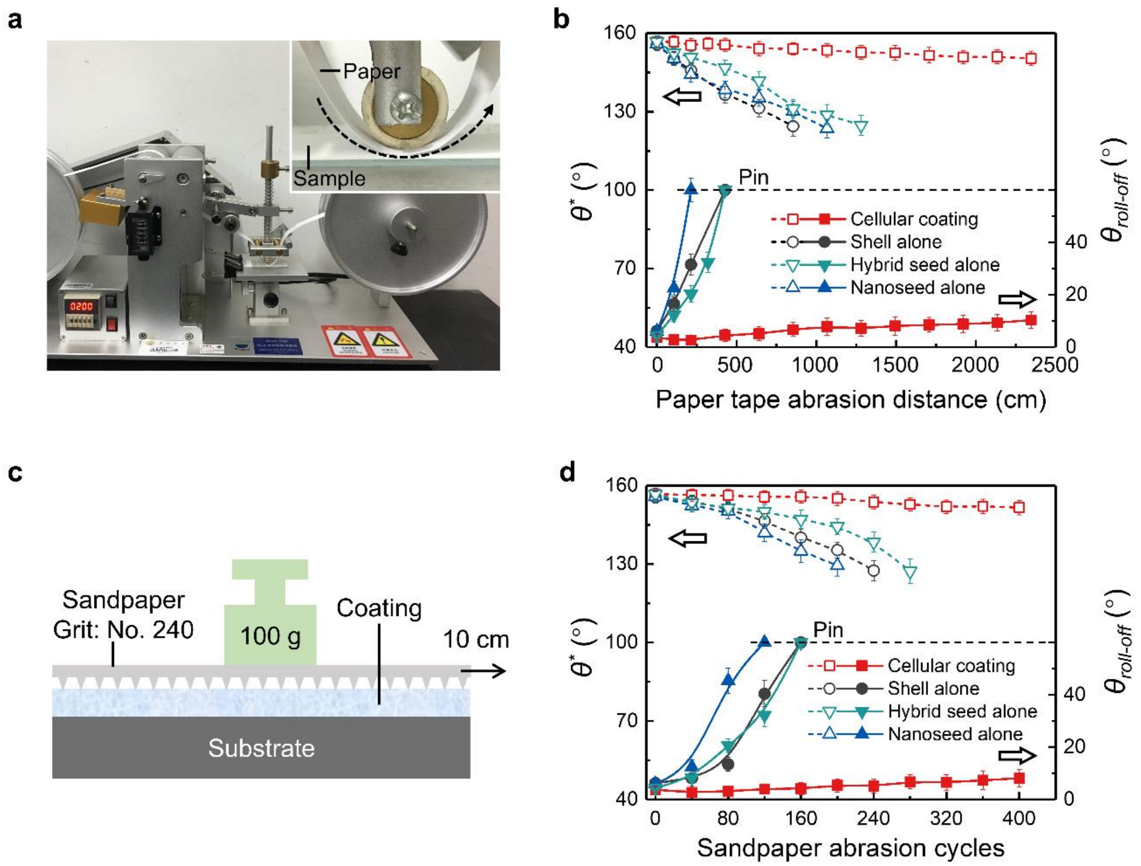

Supplementary Fig. 18 Mechanical abrasion test. a,**b**, RCA paper tape abrasion under 21-kPa load. (**a**) Optical photograph of the RCA paper tape abrasion tester, inset: a closer view of the coating under abrasion tape. (**b**) Evolution of the contact angles and roll-off angles of different coatings along with the abrasion distance. **c**,**d**, Sandpaper abrasion. (**c**) Optical image showing the abrasion process. (**d**) Evolution of the contact angles and roll-off angles of different coatings along with the abrasion cycles. The errors represent the standard deviations from at least five independent experiments.

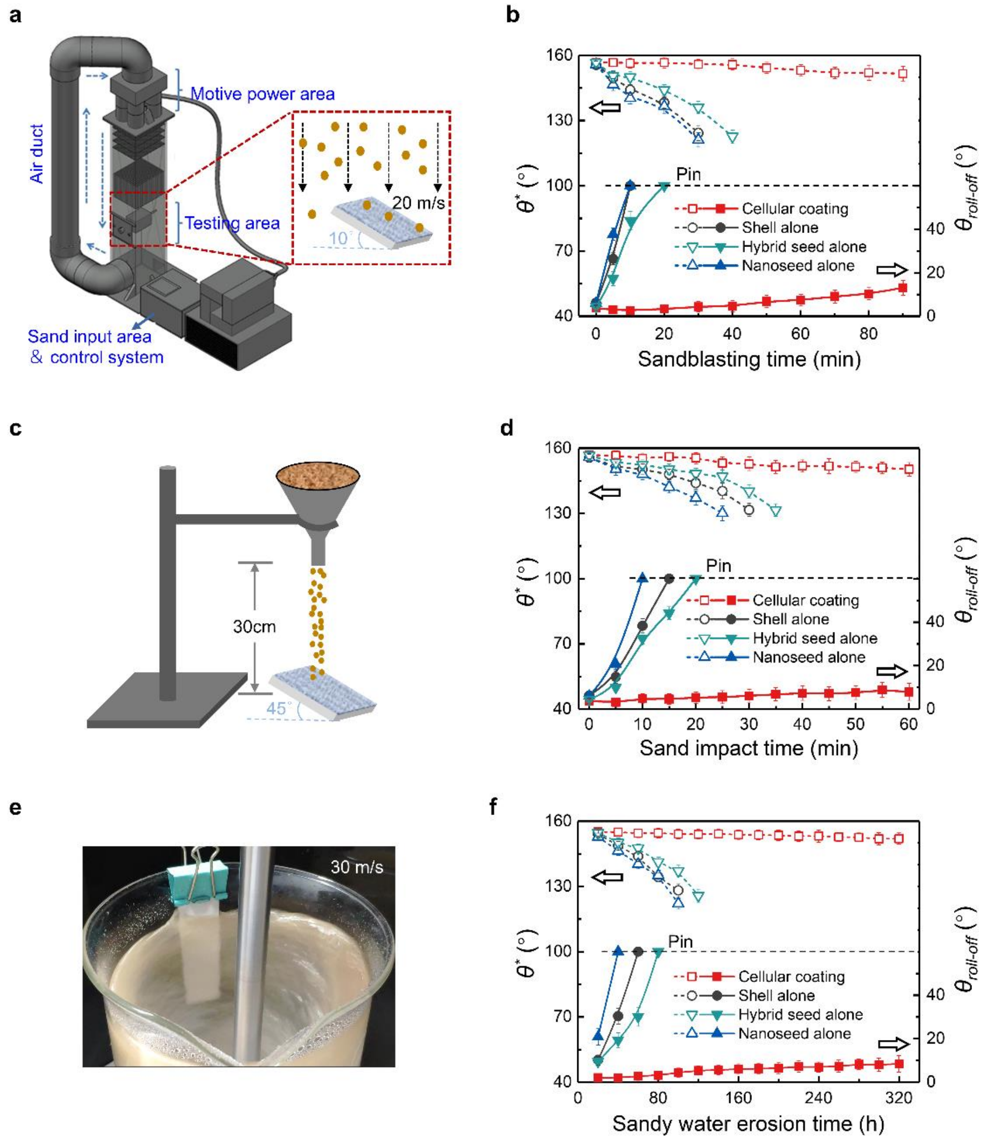

Supplementary Fig. 19 Mechanical crush test. a,**b**, Sandblasting test. (**a**) Schematic illustration of the sandblasting apparatus and procedure. (**b**) Change in the contact angles and roll-off angles of different coatings along with the sandblasting time (wind speed: 20 m s^{-1}, sand concentration: 2.2 g m^{-3}, sand size: 100–250 μm). **c**,**d**, Sand impact test. (**c**) Schematic illustration of the test. (**d**) Change in the contact angles and roll-off angles of different coatings along with the sand impacting time. **e**,**f**, Sandy water erosion test. (**e**) Schematic illustration of sandy water erosion. (**f**) Change in the contact angles and roll-off angles of different coatings during sandy water erosion (flow speed ~30 m s^{-1}, sand concentration: 4 g L^{-1}). The errors represent the standard deviations from at least five independent experiments.

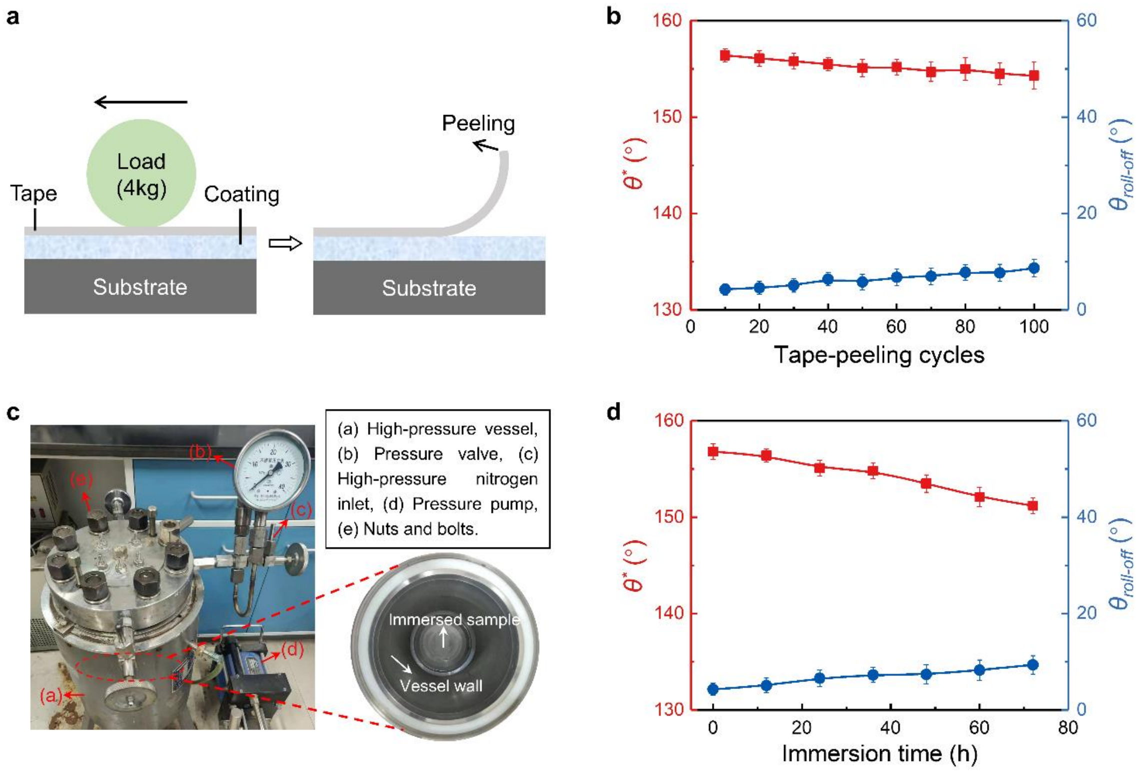

Supplementary Fig. 20 Substrate adhesion tested by tape peeling and high-pressure hydrostatic immersion. a, Schematic illustration of the tape-peeling test. **b**, Change in the contact angles and roll-off angles of the cellular coating during 100 cycles of tape-peeling. **c**, The optical photograph of the setup of the high-pressure vessel. **d**, Change in the contact angles and roll-off angles of the cellular coating during immersion under 6 MPa hydrostatic pressure. The errors represent the standard deviations from at least five independent experiments.

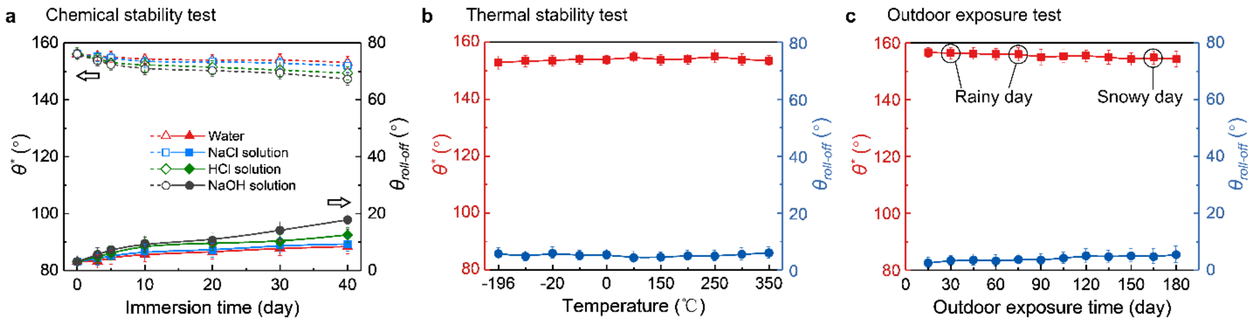

Supplementary Fig. 21 Chemical and environmental stability. a, Change in the contact angles and roll-off angles of cellular coatings along with the immersion time in the water, 3.5 wt.% NaCl solution, NaOH solution (pH ~9), and HCl solution (pH ~ 5). **b**, Change in the contact angles and roll-off angles of cellular coatings after treatment at a temperature ranging from -196 to 350 °C. The treatment time at each temperature point is 2 h. **c**, Change in the contact angles and roll-off angles of cellular coatings along with the outdoor exposure time. The cellular coating was exposed to sunlight illumination, rain impact, freezing by snow and ice, and dust contamination. The errors represent the standard deviations from at least five independent experiments.

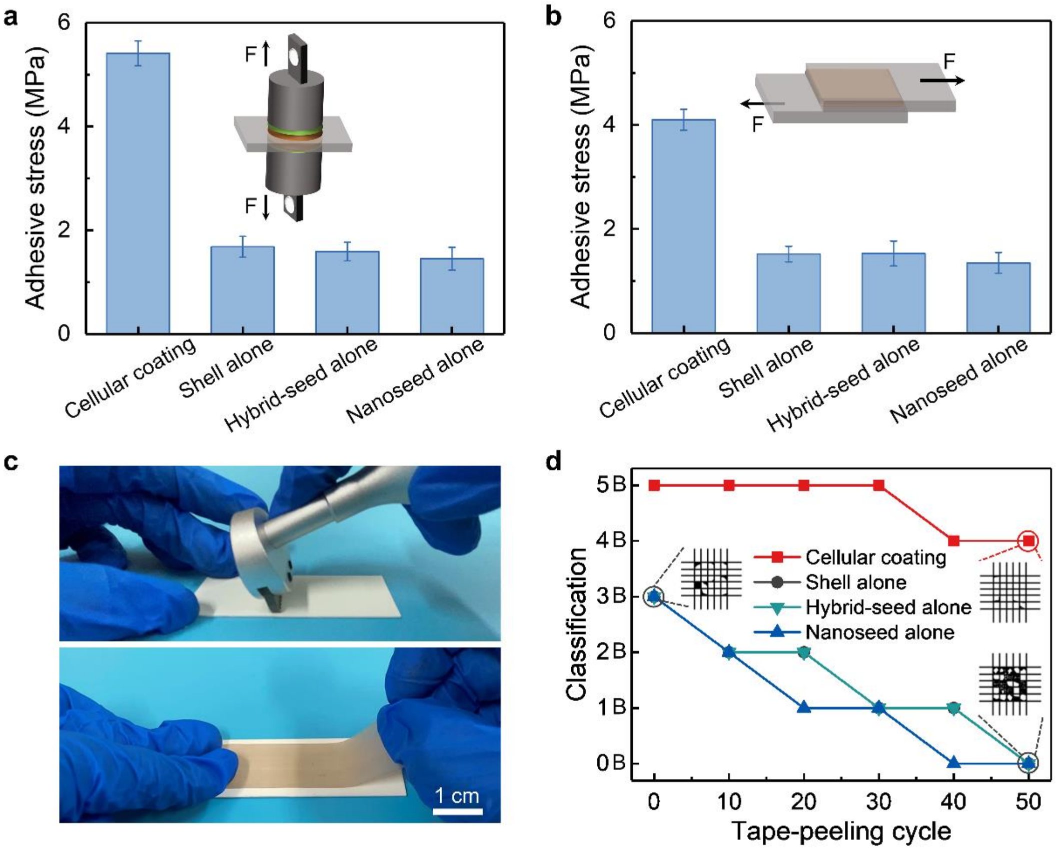

Supplementary Fig. 22 Substrate adhesion of the superhydrophobic cellular coating. a,**b**, Normal (a) and tangential (b) adhesive strength of different coatings. **c**, Cross hatch tape peel test according to the ASTM D3359-17 standard. **d**, Adhesion classification of different coatings. The errors represent the standard deviations from at least three independent experiments.

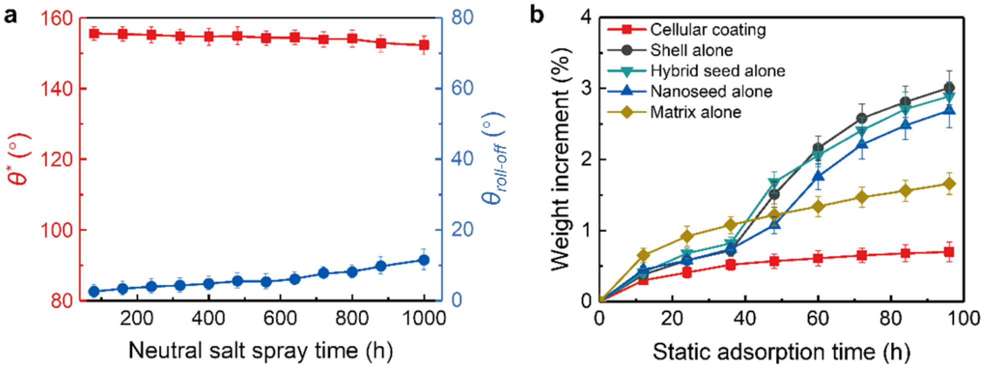

Supplementary Fig. 23 Vapor imperviousness (according to ASTM B117 standard). a, Change in the water repellence of the cellular coating during salt spraying corrosion. **b,** The moisture absorption of cellular coating in high humidity. The other coatings were used for comparison. Test conditions: relative humidity ~90%, temperature ~35 °C. The errors represent the standard deviations from at least five independent experiments.

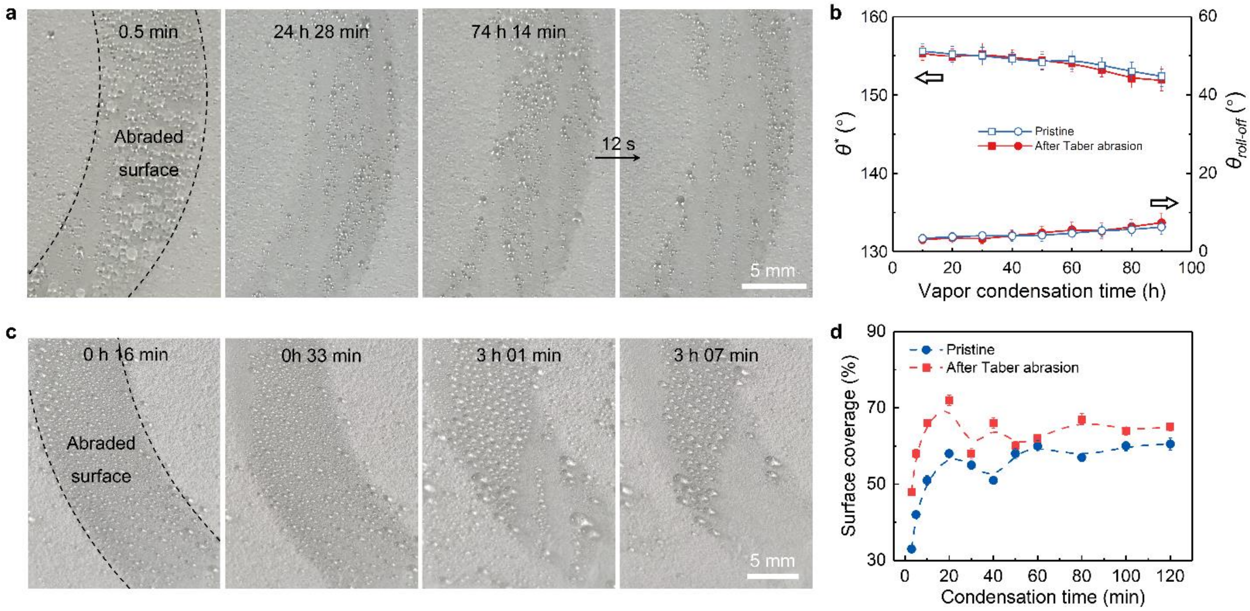

Supplementary Fig. 24 Condensation on the cellular coatings after 200-cycle Taber abrasion under 1-kg load. a,**b**, Condensation at high temperature (vapor temperature ~100 °C, coating temperature ~25 °C). (**a**) The optical photographs show the timely removal of condensate on the pristine and abraded coatings. (**b**) Change in the water repellence of the coatings during vapor condensation. **c**,**d**, Condensation at low temperature (vapor temperature ~25 °C, coating temperature ~2 °C). (**c**) The optical photographs show the timely removal of condensate on the pristine and abraded coatings. (**d**) Evolution of surface coverage during 2-h condensation. The errors represent the standard deviations from at least three independent experiments.

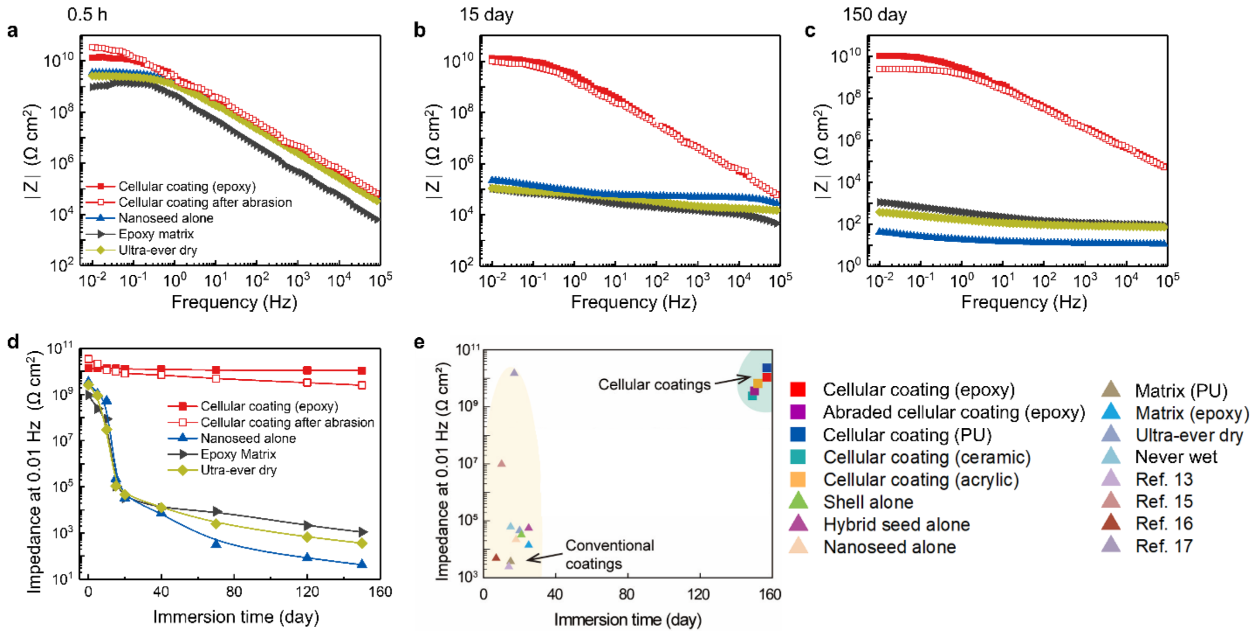

Supplementary Fig. 25 Long-term immersion and electrochemical analysis in NaCl corrosives. a-c, Bode plot of the coated steel (Q235) immersed in 3.5 wt.% NaCl solution for 0.5 h (**a**), 15 d (**b**) and 150 d (**c**). **d**, Time-dependent impedance modulus at the low frequency (0.01Hz amplitude-frequency) of different coatings. **e**, Comparison of the anti-corrosion capability of different coatings. The green area represents cellular coating and the light-yellow region refers to conventional coatings. The impedances at 0.01-Hz amplitude-frequency of our coatings are 5 orders of magnitude higher than the controls only after 15-day immersion. The impedances of conventional coatings sharply decreased within the 15-day immersion. Besides, cellular coating maintained anti-corrosion for more than 150 days, which is 10 folds of the existing reports.

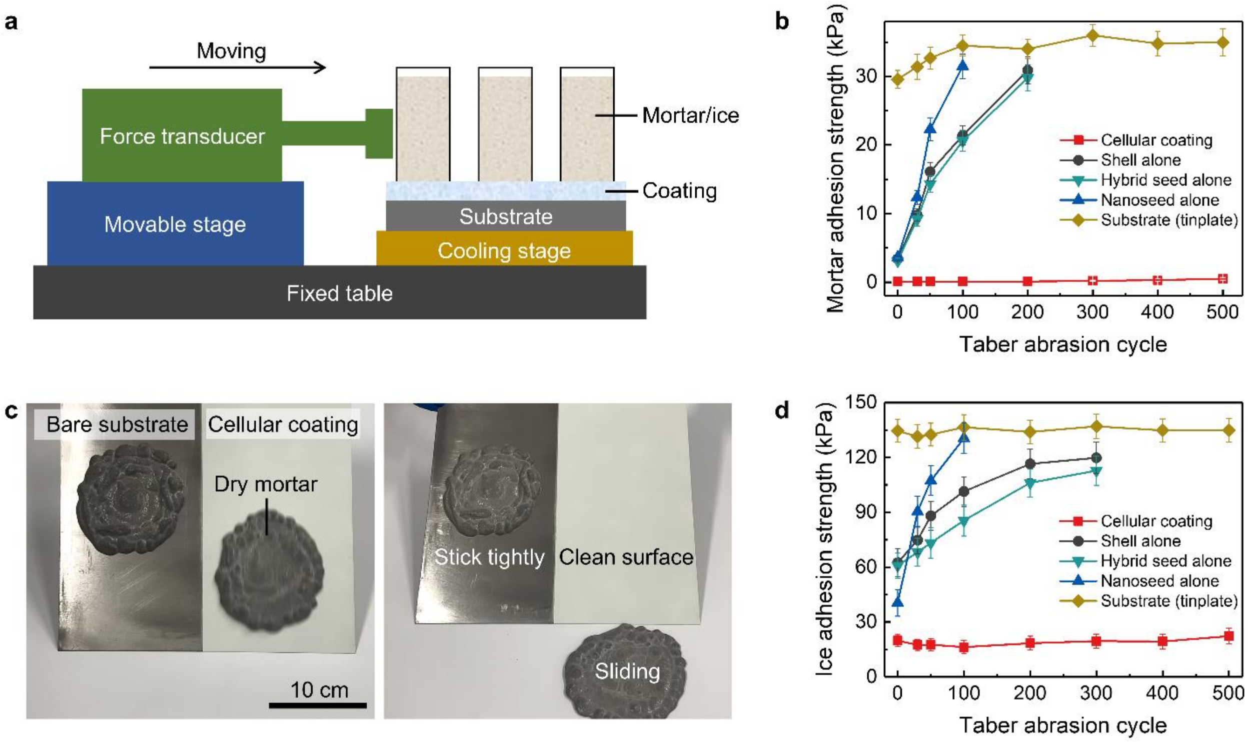

Supplementary Fig. 26 Mortar and ice shedding test. a, Schematic illustration of the adhesion strength measurement. **b**, Change in the ice adhesion strength of different coatings along with repeated Taber abrasion under 1-kg load. **c**, Optical photographs showing the self-removal of solidified mortar on cellular coating. To contrary, the mortar is tightly stuck to the bare substrate. **d**, Change in the mortar adhesion strength of different coatings along with repeated Taber abrasion under 1-kg load. The errors represent the standard deviations from at least five independent experiments.

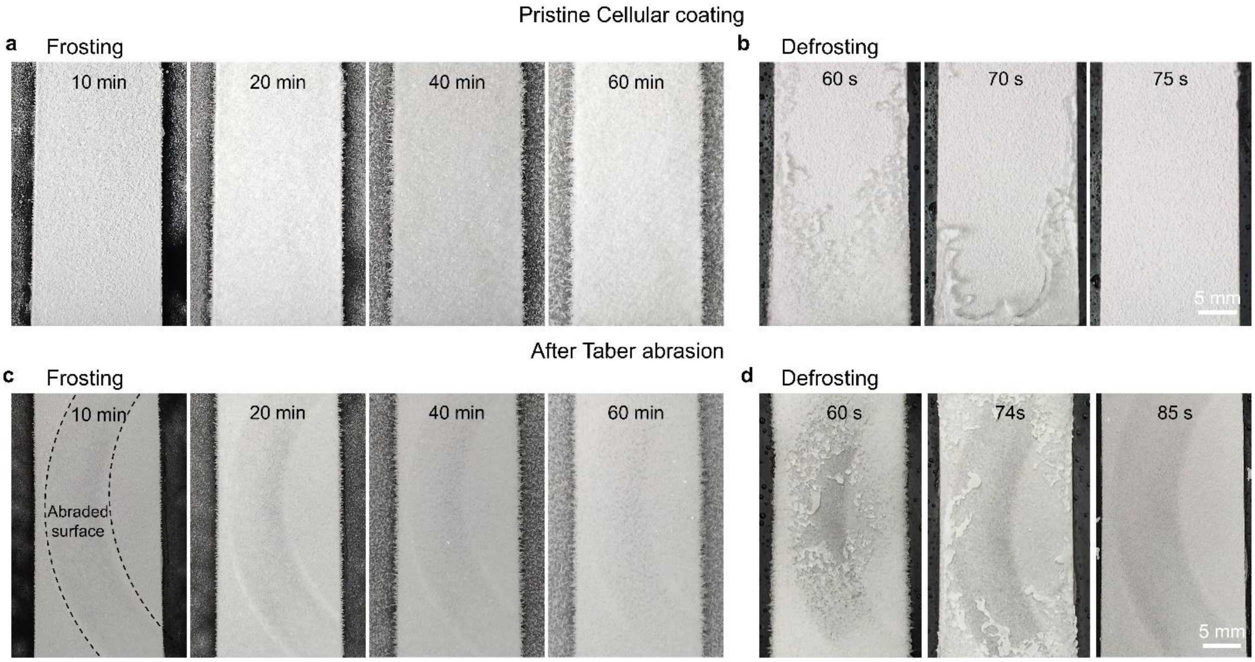

Supplementary Fig. 27 Solid shedding test. The frost sheet can be rapidly shed off from the coating even after 200-cycle Taber abrasion damage under the 1-kg load. a,**b**, Time-dependent photographs showing the frosting (**a**) and defrosting (**b**) process on pristine cellular coating. **c**,**d**, Time-dependent photographs showing the frosting (**c**) and defrosting (**d**) process on abraded cellular coating. After defrosting, both the pristine and abraded cellular surfaces were maintained dry without any residual water, exhibiting excellent frosting resistance.

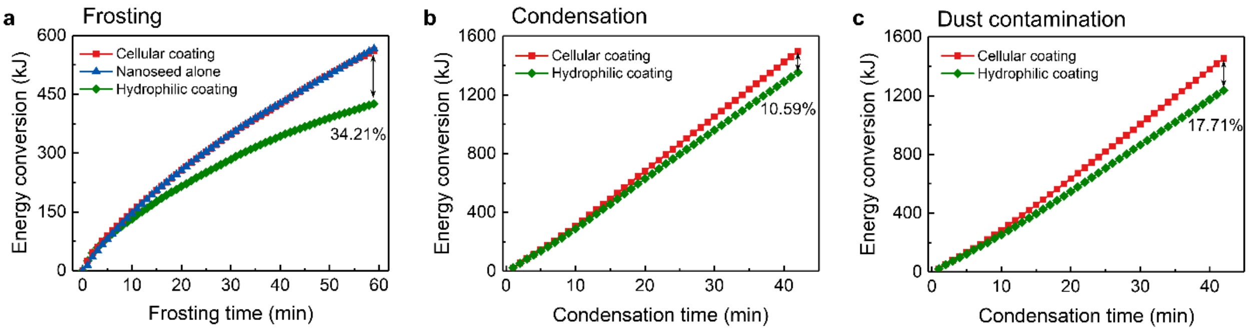

Supplementary Fig. 28 Energy conversion improvement under different operating conditions. a, frost coverage, **b**, condensate coverage, and **c**, dust contamination.

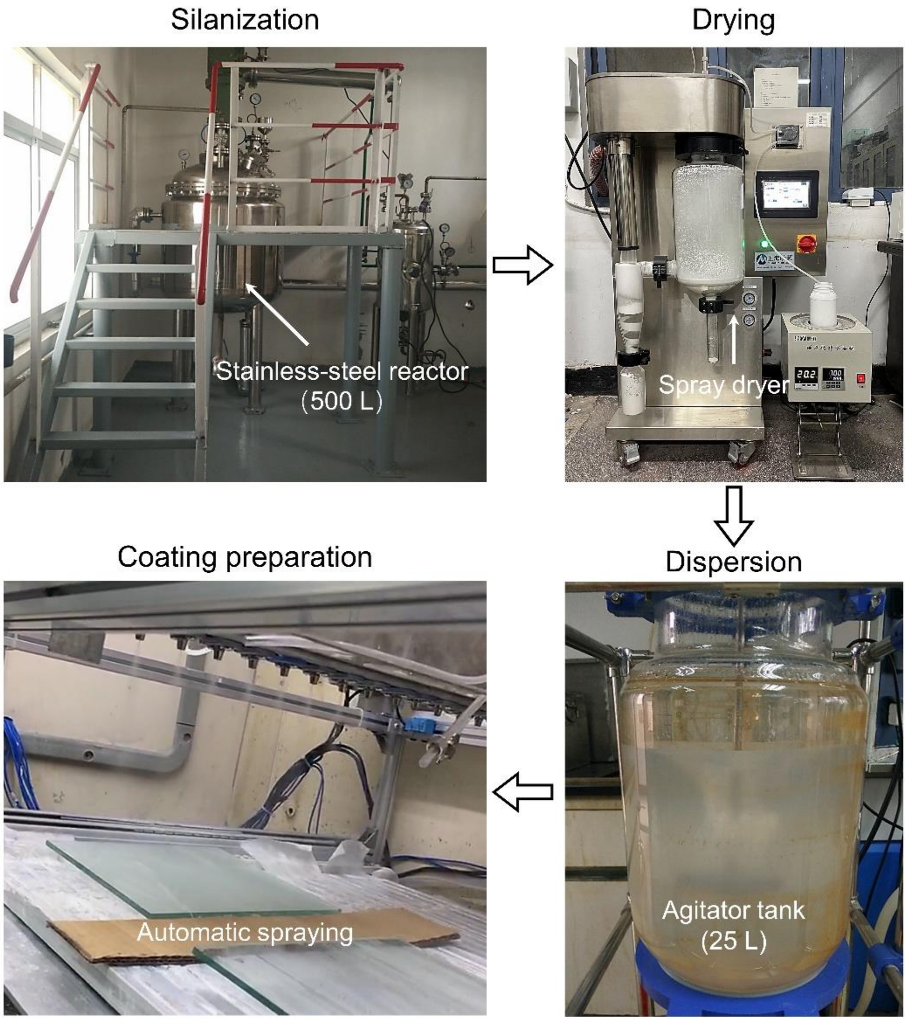

Supplementary Fig. 29 Optical photographs of the large-scale preparation of the cellular coatings. As a demonstration of scalable fabrication, we developed a serial line allowing the production of 500 L of coating dispersion solution one time,

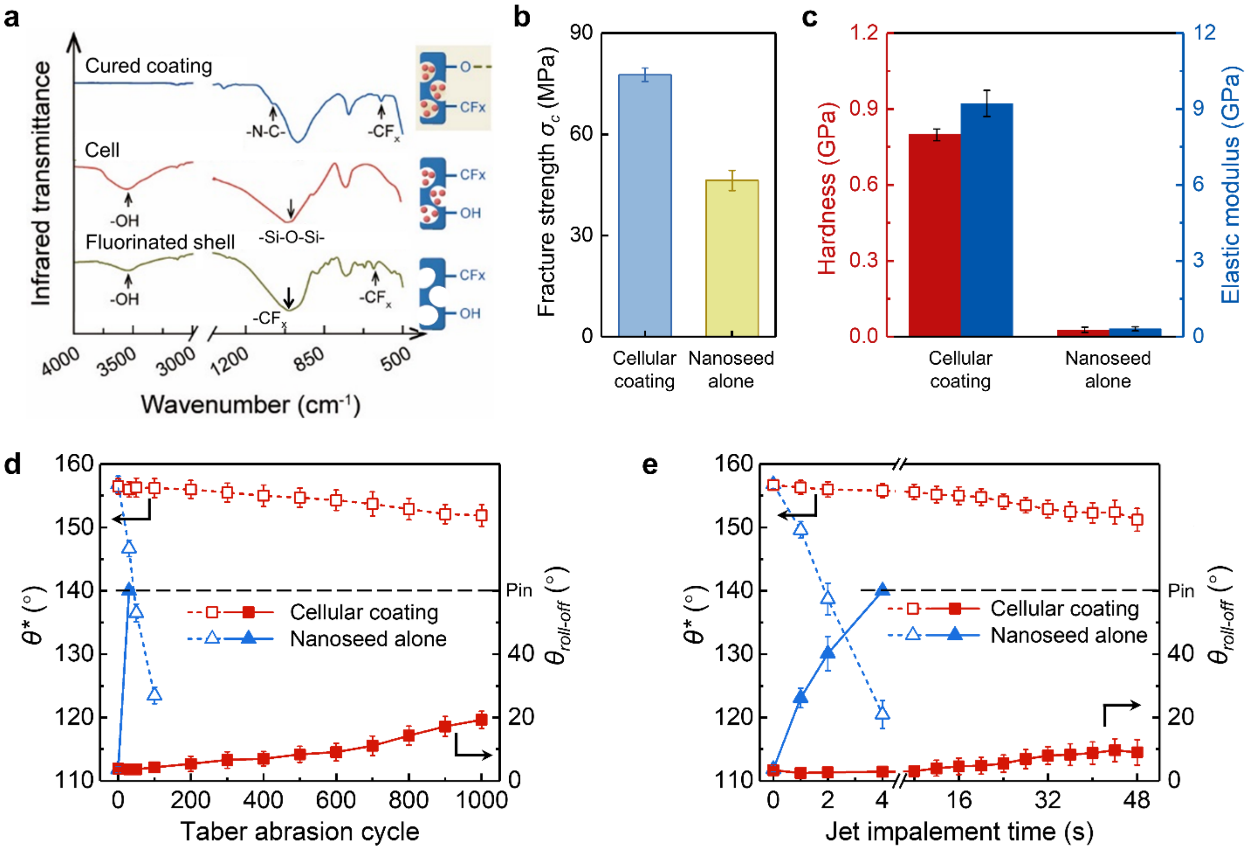

Supplementary Fig. 30 Fabrication and mechanical properties of the fluorinated cellular coatings. In such coatings, the surfaces of the nanoseeds and shells were modified with 1H,1H,2H,2H-perfluoro- decyltriethoxysilane (PFDTS) instead of OTS, and the matrix was fluoroethylene vinyl ether resin (FEVE). The nanoseed-alone coating was used for comparison. **a**, FTIR spectra of the fluorinated shells, cells, and cellular coating after curing. **b**, Fracture strength of the coatings. **c**, Hardness and elastic modulus of the coatings. **d**, Evolution of water contact angles and roll-off angles of the coatings during Taber abrasion under 1-kg load. **e**, Evolution of water contact angles and roll-off angles of the coatings along with the water jet impact time (jet velocity ~40 m s^{-1}, *We* ~44444). The errors represent the standard deviations from at least three independent experiments.

Supplementary Table

Supplementary Table 1. Summary of the existing strategy for improving the durability of superhydrophobic coatings

Bulk phase design				
Author	Method	Materials	Durability test	Test result
Yao Lu et al[1]	Introducing adhesives	TiO_2, the spray adhesive (EVO-STIK), double-sided tapes (Niceday)	Sandpaper abrasion<br>Sandpaper: grit no. 220<br>Load: 100 g	Bearing 40 cycles
Mingming Liu et al[18]	Introducing adhesives	TiO_2, aluminum phosphate	Sandpaper abrasion<br>Sandpaper: grit no. 1000<br>Load: 50 g	Bearing 100 cycles
Chaoyi Peng et al[2]	Softening peak stress	PTFE, Krytox, epoxy, fluorinated amine	Taber abrasion<br>Load: 250 g	Bearing 80-90 cycles
Wentao Cao et al[19]	Softening peak stress	$Ti_3C_2T_x$ MXene, polydimethylsiloxane	Sandpaper abrasion<br>Sandpaper: grit no. 800<br>Load: 200 g	Bearing 10 cycles
Xu Deng et al[3]	Elevating coating modulus and rigidity	SiO_2, soot particles	Falling sand impact<br>falling height: 40 cm	Bearing 20-g sand impact
Jiefeng Gao et al[20]	Elevating coating modulus and rigidity	Commercial thermoplastic polyurethanes, SiO_2, graphene oxide	Falling sand impact<br>falling height: 20 cm	Bearing 30-g sand impact
Jinlong Song et al[21]	Elevating coating modulus and rigidity	Concrete	Sandpaper abrasion<br>Sandpaper: grit no. 360<br>Load: 1100 Pa	Bearing 30 cycles
Chunyan Cao et al[22]	Self-healing	Bis-aminopropyl-terminated polydimethylsiloxane, dopamine hydrochloride, isophoronediisocyanate, SiO_2	Sandpaper abrasion<br>Sandpaper: grit no. 220<br>Load: 1 kg	Bearing 50 cycles
Hua Zhou et al[23]	Self-healing	Polytetrafluoroethylene nanoparticle, Zonyl321 surfactant, perfluorodecyltriethoxysilane	Falling sand impact<br>falling height: 100 cm	Bearing 15-min impact
Lihua Jiang et al[24]	Using hierarchical roughness and adhesives	SiO_2, diatomite, PDMS, KH-570, organosilane	Sandpaper abrasion<br>Sandpaper: grit no. 360<br>Load: 200 g	Bearing 17 cycles
Interfacial design				
Author	Armor structure	Materials	Durability test	Test result
Dehui Wang et al[25]	Cavity	Silicon	Taber abrasion<br>Load: 250 g	Bearing 250 cycles

Vitaliy Kondrashov et al[26]	Microcone	Silicon	Wear tests by a rheometer (MCR Anton Paar GmbH, Austria)	Bearing shear force of 20 N
Kim Jeong-Gil et al[27]	Crater-like structure<br>Needle-like structure	Polyurethane acrylate	Simulated stress distribution under an equivalent loading of 4 N	The crater-like structure bearing 1.23-1.34 MPa force.<br>The needle-like structure bearing 44.8-50.4 MPa force
Ludmila B. Boinovich et al[28]	Hierarchical structure	Aluminum-magnesium alloy	Oscillating sand abrasion (ASTM F735)	Bearing 30 min
Yong Chae Jung et al[29]	Pillar	Silicon	Waterfall test	Bearing waterfall with 25 kPa for 20 min

Supplementary Table 2. A summary of the durability tests

Classification	Test method	Standard/ Reference	Test conditions	Results	
				Cellular coating	Controls
Mechanical (i.e., abrasion, crush, adhesion, hydrostatic pressure)	Taber abrasion (Fig. 3a-c, and Supplementary Fig. 15)	ASTM D4060	Abrasive wheel: CS-10, Load: 1 kg<br>Coating thickness: 80 μm<br>Substrate: circular glass plate<br>Diameter: 100 mm	Bearing 1000-cycle abrasion<br>High wear resistance: index ~8.4	Bearing 25-cycle abrasion<br>Low wear resistance: index ~0.3
	RCA abrasion (Supplementary Fig. 18a,b)	ASTM 2357-04	Paper: width ~0.6875 inch<br>Load: 12 kPa<br>Coating thickness: 80 μm<br>Substrate: rectangle glass plate<br>Size: 75 mm × 25 mm<br>(the following samples were the same otherwise specially noted)	Bearing 2400-cm abrasion	Bearing 240-cm abrasion
	Sandpaper abrasion (Supplementary Fig. 18c,d)	Y. Lu, Science, 2015[1]	Grit: No. 240<br>Load: 100 g	Bearing 400-cycle abrasion	Bearing 40-cycle abrasion
	High-speed jet impact (Fig. 3d,e and Supplementary Figs. 16 and 17)	C. Peng, Nat. Mater. 2018[2]	Jet speed: ~40 m s^{-1}<br>*We*: ~44444	Bearing 72-s impact<br>Regeneration via gentle abrasion	Bearing 2-s impact
	Sandblasting (Supplementary Fig. 19a,b)	GBJ 150.12A	SiO_2 sand: 100–250 μm<br>Wind speed: 20 m s^{-1}<br>Sand content: 2.2 g m^{-3}	Bearing 90-min sandblasting	Bearing 5-min sandblasting
	Falling sand impact (Supplementary Fig. 19c,d)	X. Deng, Science, 2011[3]	SiO_2 sand: 100–250 μm<br>Falling height: 30 cm<br>Falling speed: 40 g min^{-1}	Bearing 60-min impact	Bearing 5-min impact
	Sandy water erosion (Supplementary Fig. 19e,f)	Designed for mimicking sandy water wash over a ship surface	SiO_2 sand: 100–250 μm<br>Flow speed: ~ 30 m s^{-1}<br>Sand content: 4 g L^{-1}	Bearing 320-h erosion	Bearing 40-h erosion

	Tape-peeling (Supplementary Fig. 20a,b)	ASTM D3359-17	3M^{TM} VHB tape<br>Load: 4 kg	Bearing 100-cycle tape-peeling
	Immersion under high hydrostatic pressure (Supplementary Fig. 20c,d)	Mimicking the deep-sea environment (~612 m)	Saltwater: 3.5 wt.%<br>Hydrostatic pressure: 6 MPa	Bearing 72-h high hydrostatic pressure
Chemical	Chemical solution immersion (Supplementary Fig. 21a)	X. Zhang, J. Mater. Chem. A, 2016[4]	Pure water<br>Saltwater: 3.5 wt.%<br>HCl solution (pH: 5)<br>NaOH solution (pH: 9)	Bearing 40-day immersion
Thermal	Thermal stability test (Supplementary Fig. 21b)	S. Pan, Nat. Mater. 2018[5]	1) From -196 °C (liquid nitrogen immersion) to 350 °C (oven heating)<br>2) Long-term baking at 200 °C	1) After 2-h treatment:<br>$\theta^* > 150°$<br>$\theta_{roll\text{-}off} < 10°$<br>2) Bearing 40-day baking at 200 °C:
Outdoor	Environmental stability test (Supplementary Fig. 21c)	S. Foorginezhad, Appl. Surf. Sci. 2019[30]	Sunlight illumination,<br>Rain impact<br>Freeze by snow and ice<br>Dust contamination	Bearing 180-day outdoor exposure
Vapor permeation and mechanical abrasion	Taber abrasion and vapor treatment (Supplementary Fig. 24a,b)	Mimicking synergistic damage in real-world applications	1)<br>Taber abrasion<br>Wheel: CS-10<br>Load: 1 kg<br>2)<br>Vapor treatment<br>Temperature: 100 °C<br>Relative humidity: 90%	Bearing 200-cycle abrasion & 90-h vapor treatment
	Taber abrasion and low-temperature condensation (Supplementary Fig. 24c,d)		1)<br>Taber abrasion<br>Wheel: CS-10<br>Load: 1 kg<br>2)<br>Low-temperature condensation<br>Temperature: 2 °C<br>Relative humidity: 70%	After 200-cycle abrasion & 12-h test:<br>dropwise condensation and easy removal of the droplet by self-jumping and gravity;<br>droplet average radius: 18 μm

	High-humidity environment treatment (Supplementary Fig. 23)		Relative humidity: 90%<br>Temperature: 35 °C	Bearing 1000-h treatment<br>Moisture absorption: 0.7 wt.%	Bearing 72-h treatment<br>Moisture absorption: 2.7-3 wt.%
Solid adhesion and mechanical damages	Ultra-low temperature frosting and Taber abrasion (Supplementary Fig. 27)	Mimicking synergistic damage in real-world applications	1)<br>Taber abrasion<br>Wheel: CS-10<br>Load: 1 kg<br>2)<br>Ultralow temperature frosting<br>Temperature: -20 °C<br>Relative humidity: 70%	After 200-cycle abrasion & 1 cycle of frosting and defrosting:<br>dry surface without any residual water	
	Dry Mortar slurry adhesion and Taber abrasion (Fig. 4c and Supplementary Fig. 26a-c)		1)<br>Taber abrasion<br>Wheel: CS-10<br>Load: 1 kg<br>2)<br>Mortar (200 g) dry on the surface	Mortar adhesion strength after 500-cycle abrasion: 0.9 kPa<br>Dry mortar falls off by gravity (tilt angle ~30°)	Mortar adhesion strength after 100-cycle abrasion: 56.5 kPa<br>Dry mortar sticks to tinplate substrate
	Mortar slurry adhesion and mechanical damages (Supplementary Movie 4)		1)<br>Steel-wool abrasion<br>Average force: 7.6 N<br>2)<br>Sandpaper abrasion<br>Average force: 6.6 N<br>3)<br>Screwdriver scratch<br>Average force: 4.8 N<br>4)<br>Mortar slurry on the surface<br>Pressure: 4 kPa<br>Duration: 20 min	Mortar slurry rolls off by gravity (tilt angle ~30°)	Mortar adheres to tinplate substrate
	Icing and Taber abrasion (Supplementary Fig. 26d)		1)<br>Taber abrasion<br>Wheel: CS-10<br>Load: 1 kg<br>2)<br>Icing	Ice adhesion strength after 500-cycle abrasion: 22.3 kPa	Ice adhesion strength after 100-cycle abrasion: 130.5 kPa

Temperature: -20 °C

Relative humidity: 70%

Supplementary Table 3. Differences between the large-scale and in-lab preparation

Preparation step	Large-scale preparation	In-lab preparation
Silanization	• Mix **14.4 kg** of nanoseeds, **320 L** of ethanol, **32 L** of deionized water, and **16 L** of ammonia hydroxide by mechanical stir (**1000 r min^{-1}**) for **4 h**. Silanize diatomite shells using the same method.<br>• Add **1.92 L** of OTS and stirred at 50 °C for **48 h**.	• Mix **36 g** of nanoseeds, **800 mL** of ethanol, **80 mL** of deionized water, and **40 mL** of ammonia hydroxide by mechanical stir (**600 r min^{-1}**) for **0.5 h**. Silanize diatomite shells using the same method.<br>• Add **4.8 mL** of OTS and stirred at 50 °C for **24 h**.
Drying	• **Spray drying**	• **Freeze-drying in a vacuum environment (~0.04 MPa) at -30 °C for 24 h.**
Dispersion	• Load the nanoseeds (**0.6 kg**) to the shell (**2 kg**) by stirring their mixture in butyl acetate (**15 kg**) for **1 h**.<br>• Suspend the cells into **6.8-kg** epoxy matrix by stirring for **3 h**.	• Load the nanoseeds (**0.6 g**) to the shell (**2 g**) by stirring their mixture in butyl acetate (**15 g**) for **10 min**.<br>• Suspend the cells into **6.8-g** epoxy matrix by stirring for **0.5 h**.
Coating preparation	• **Automatically spray**	• **Manually spray**

Supplementary References

1. Lu, Y., Sathasivam, S., Song, J., Crick, C. R., Carmalt, C. J. & Parkin, I. P. Robust self-cleaning surfaces that function when exposed to either air or oil. *Science* **347**, 1132-1135 (2015). doi:10.1126/science.aaa0946
2. Peng, C., Chen, Z. & Tiwari, M. K. All-organic superhydrophobic coatings with mechanochemical robustness and liquid impalement resistance. *Nat. Mater*. **17**, 355-360 (2018). doi:10.1038/s41563-018-0044-2
3. Deng, X., Mammen, L., Butt, H. J. & Vollmer, D. Candle soot as a template for a transparent robust superamphiphobic coating. *Science* **335**, 67-69 (2012). doi:10.1126/science.1207115
4. Zhang, X., Zhu, W., He, G., Zhang, P., Zhang, Z. & Parkin, I. P. Flexible and mechanically robust superhydrophobic silicone surfaces with stable Cassie–Baxter state. J. Mater. Chem. A **4**, 14180–14186 (2016). doi:10.1039/c6ta06493k
5. Pan, S., Guo, R., Bjornmalm, M., Richardson, J. J., Li, L., Peng, C., Bertleff-Zieschang, N., Xu, W., Jiang, J. & Caruso, F. Coatings super-repellent to ultralow surface tension liquids. *Nat. Mater.* **17**, 1040-1047 (2018). doi:10.1038/s41563-018-0178-2
6. Kresse, G. & Furthmüller, J. Efficient iterative schemes for ab initiototal-energy calculations using a plane-wave basis set. *Phys. Rev. B: Condens. Matter Mater. Phys.* **54**, 11169−11186 (1996). doi: 10.1103/PhysRevB.54.11169
7. Perdew, J. P., Burke, K. & Ernzerhof, M. Generalized gradient approximation made simple. *Phys. Rev. Lett.* **78**, 3865 (1996). doi:10.1103/PhysRevLett.77.3865
8. Tkatchenko, A. & Scheffler, M. Accurate molecular Van Der Waals interactions from ground-state electron density and free-atom reference data. *Phys. Rev. Lett.* **102**, 073005 (2009). doi:10.1103/PhysRevLett.102.073005
9. Monkhorst, H. J. & Pack, J. D. Special points for Brillouin-zone integrations. *Phys. Rev. B* **13**, 5188 (1976). doi:10.1103/PhysRevB.13.5188
10. Zhang, Y., Zhang, L., Xiao, Z., Wang, S. & Yu, X. Fabrication of robust and repairable superhydrophobic coatings by an immersion method. *Chem. Eng. J.* **369**, 1-7 (2019). doi:10.1016/j.cej.2019.03.021
11. Xiao, Z., Zhu, H., Wang, S., Dai, W., Luo, W., Yu, X. & Zhang, Y. Multifunctional superwetting composite coatings for long-term anti-icing, air purification, and oily water separation. *Adv. Mater. Interfaces* **7**, 2000013 (2020). doi:10.1002/admi.202070041

12. Sun, Y. & Guo, Z. A scalable, self-healing and hot liquid repelling superamphiphobic spray coating with remarkable mechanochemical robustness for real-life applications. *Nanoscale* **11**, 13853 (2019). doi:10.1039/C9NR02893E
13. Li, D., Wang, H., Liu, Y., Wei, D. & Zhao, Z. Large-scale fabrication of durable and robust superhydrophobic spray coatings with excellent repairable and anti-corrosion performance. *Chem. Eng. J.* **367**, 169 (2019). doi:10.1016/j.cej.2019.02.093
14. Kong, L., Kong, X., Ji, Z., Wang, X. & Zhang, X. Large-scale fabrication of a robust superhydrophobic thermal energy storage sprayable coating based on polymer nanotubes. *ACS Appl. Mater. Interfaces* **12**, 49694–49704 (2020). doi:10.1021/acsami.0c15531
15. Zhang, X., Jiang, F., Chen, R., Chen, Y. & Hu, J. Robust superhydrophobic coatings prepared by cathodic electrophoresis of hydrophobic silica nanoparticles with the cationic resin as the adhesive for corrosion protection. *Corros. Sci.* **173**, 108797 (2020). doi:10.1016/j.corsci.2020.108797
16. Zhang, X., Chen, Y. & Hu, J. Robust superhydrophobic SiO_2/polydimethylsiloxane films coated on mild steel for corrosion protection. *Corros. Sci.* **166**, 108452 (2020). doi:10.1016/j.corsci.2020.108452
17. Zhao, X., Wei, J., Li, B., Li, S., Tian, N., Jing, L. & Zhang, J. A self-healing superamphiphobic coating for efficient corrosion protection of magnesium alloy. *J. Colloid Interf. Sci.* **575**, 140-149 (2020). doi:10.1016/j.jcis.2020.04.097
18. Liu, M., Hou, Y., Li, J., Tie, L., Peng, Y. & Guo, Z. Inorganic adhesives for robust, self-healing, superhydrophobic surfaces. *J. Mater. Chem. A* **5**, 19297-19305 (2017). doi:10.1039/c7ta06001g
19. Cao, W., Feng, W., Jiang, Y., Ma, C., Zhou, Z., Ma, M., Chen, Y. & Chen, F. Two-dimensional MXene-reinforced robust surface superhydrophobicity with self-cleaning and photothermal-actuating binary effects. *Mater. Horiz.* **6**, 1057-1065 (2019). doi:10.1039/c8mh01566j
20. Gao, J., Li, B., Huang, X., Wang, L., Lin, L., Wang, H. & Xue, H. Electrically conductive and fluorine free superhydrophobic strain sensors based on SiO_2/graphene-decorated electrospun nanofibers for human motion monitoring. *Chem. Eng. J.* **373**, 298-306 (2019). doi:10.1016/j.cej.2019.05.045

21. Song, J., Zhao, D., Han, Z., Xu, W., Lu, Y., Liu, X., Liu, B., Carmalt, C. J., Deng, X. & Parkin, I. P. Super-robust superhydrophobic concrete. *J. Mater. Chem. A* **5**, 14542-14550 (2017). doi:10.1039/c7ta03526h
22. Cao, C., Yi, B., Zhang, J., Hou, C., Wang, Z., Lu, G., Huang, X. & Yao, X. Sprayable superhydrophobic coating with high processibility and rapid damage-healing nature. *Chem. Eng. J.* **392**, 124834 (2020). doi:10.1016/j.cej.2020.124834
23. Zhou, H., Wang, H., Niu, H., Zhao, Y., Xu, Z. & Lin, T. A waterborne coating system for preparing robust, self-healing, superamphiphobic surfaces. *Adv. Funct. Mater.* **27**, 1604261 (2017). doi:10.1002/adfm.201604261
24. Jiang, L., Hou, P., He, S, Han, M., Xiang, P., Xiao, T. & Tan, X. The robust superhydrophobic SiO_2/Diatomite/PDMS/KH-570/Me-MQ composite coating for self-cleaning application of building surface. *Colloid Surf. A-Physicochem. Eng. Asp.* **634**, 127936 (2022). doi:10.1016/j.colsurfa.2021.127936
25. Wang, D., Sun, Q., Hokkanen, M. J., Zhang, C., Lin, F., Liu, Q., Zhu, S., Zhou, T., Chang, Q., He, B., Zhou, Q., Chen, L., Wang, Z., Ras, R. H. A. & Deng, X. Design of robust superhydrophobic surfaces. *Nature* **582**, 55-59 (2020). doi:10.1038/s41586-020-2331-8
26. Kondrashov, V. & Rühe, J. Microcones and nanograss: toward mechanically robust superhydrophobic surfaces. *Langmuir* **30**, 4342−4350 (2014). doi:10.1021/la500395e
27. Kim, J. G., Choi, H. J., Park, K. C., Cohen, R. E., McKinley, G. H. & Barbastathis, G. Multifunctional inverted nanocone arrays for non-wetting, self-cleaning transparent surface with high mechanical robustness. *Small* **10**, 2487–2494 (2014). doi:10.1002/smll.201303051
28. Boinovich, L. B., Modin, E. B., Sayfutdinova, A. R., Emelyanenko, K. A., Vasiliev, A. L., & Emelyanenko, A. M. Combination of functional nanoengineering and nanosecond laser texturing for design of superhydrophobic aluminum alloy with exceptional mechanical and chemical properties. *ACS Nano* **11**, 10113-10123 (2017). doi:10.1021/acsnano.7b04634
29. Jung, Y. C. & Bhushan, B. Mechanically durable carbon nanotube-composite hierarchical structures with superhydrophobicity, self-cleaning, and low-drag. *ACS Nano* **3**, 4155-4163 (2009). doi:10.1021/nn901509r
30. Foorginezhad, S. & Zerafat, M. M. Fabrication of stable fluorine-free superhydrophobic fabrics for anti-adhesion and self-cleaning properties. *Appl. Surf. Sci.* **464**, 458-471 (2019). doi:10.1016/j.apsusc.2018.09.058
