# Supplementary material for: Ultra-durable superhydrophobic cellular coatings
Source: Nat Commun. 2023 Sep 23;14:5953. doi: 10.1038/s41467-023-41675-y (PMC10517967; doi:10.1038/s41467-023-41675-y)
Supplement: Supplementary file 3 — Description of Additional Supplementary Files [file 41467_2023_41675_MOESM3_ESM.pdf]

Description of Additional Supplementary Files

Supplementary Movie 1. Mechanical durability test of cellular coatings by Taber abrasion under 1-kg load. The coating maintained superhydrophobic after abrasion for 1000 cycles, which is 100 times of the conventional reports.

Supplementary Movie 2. High-speed jet impalement test. This video is divided into two parts. In Part. I, the cellular coating was treated with the water jet at a velocity of ~40 m s -1 (We ~44444), and well maintained the water repellence for 48 s. In Part II, although water jetting finally drove the failure of water repellence, we demonstrated that after gentle abrasion with sandpaper, the cellular coating regained its water repellence. This regeneration cycle can be repeated for prolonged service of cellular coating against heavy mechanical damage.

Supplementary Movie 3. Mechanical durability test of cellular coatings against human foot stamping. For the test, four human adults stepped on/off the coating for more than 1000 steps. The adults include one man (55 kg weight) wearing running shoes, one man (75 kg weight) wearing casual shoes, one woman (55 kg weight) wearing high heels, and one man (85 kg weight) wearing running shoes. The coatings maintained their superhydrophobicity after the tests.

Supplementary Movie 4. Solid shedding test. This video is divided into three parts. In Part. I, the mortar was poured onto a tinplate plate, with half surface protected by the cellular coating. After curing for 24 h, the entire solid mortar on the cellular coating fell off driven by gravity, whereas got stuck on the bare surface and hardly removed. In Part. Ⅱ, a drop of mortar slurry can bead up on cellular coating after repeated compression without a noticeable change in the receding angle, and easily rolled off at a tilt angle of 6°. In Part Ⅲ, a large amount of mortar slurry easily rolled off from the cellular coating even after being damaged with steel-wool abrasion (average force ~7.6 N), sandpaper abrasion (average force ~6.6 N), screwdriver scratching (average force ~4.8 N) and pressing (~4 kPa, duration ~20min). 45

Supplementary Movie 5. Defrosting test on cellular coating. A commercial hydrophilic coating was used for comparison. On cellular coating, the defrosting was achieved via self-peeling off of the entire frost sheet in a short time. In contrast, on the commercial control surface, the frost experienced melting, drainage, and evaporation for a long duration with 2.8-fold energy consumption of that on cellular coating.
